# Supplementary material for: The Evolution of Mitochondrial Genomes between Two Cymbidium Sister Species: Dozens of Circular Chromosomes and the Maintenance and Deterioration of Genome Synteny
Source: Genes (Basel). 2023 Apr 3;14(4):864. doi: 10.3390/genes14040864 (PMC10137497; doi:10.3390/genes14040864)

Fig. S1 | Plant materials used in this study. A) The morphology of *C. lancifolium*. B) Leaves of a *C. lancifolium* seedling were used for sequencing. C) The morphology of *C. macrorhizon*. D) Scapes and rhizomes of a *C. macrorhizon* seedling were used for sequencing.

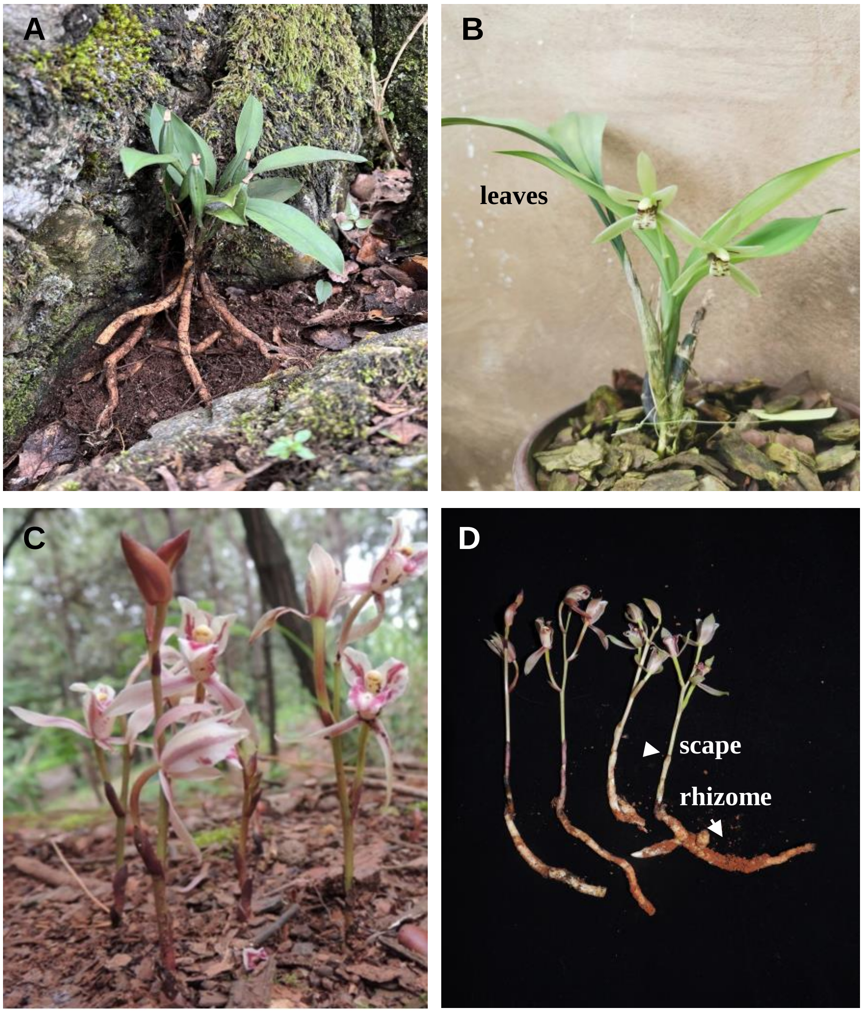

Fig. S2 | Diagrams of Illumina-based draft assemblies. A) *C. lancifolium* mitogenome assembly. B) *C. macrorhizon* mitogenome assembly. Contigs with high read coverage (>50x) are highlighted in green.

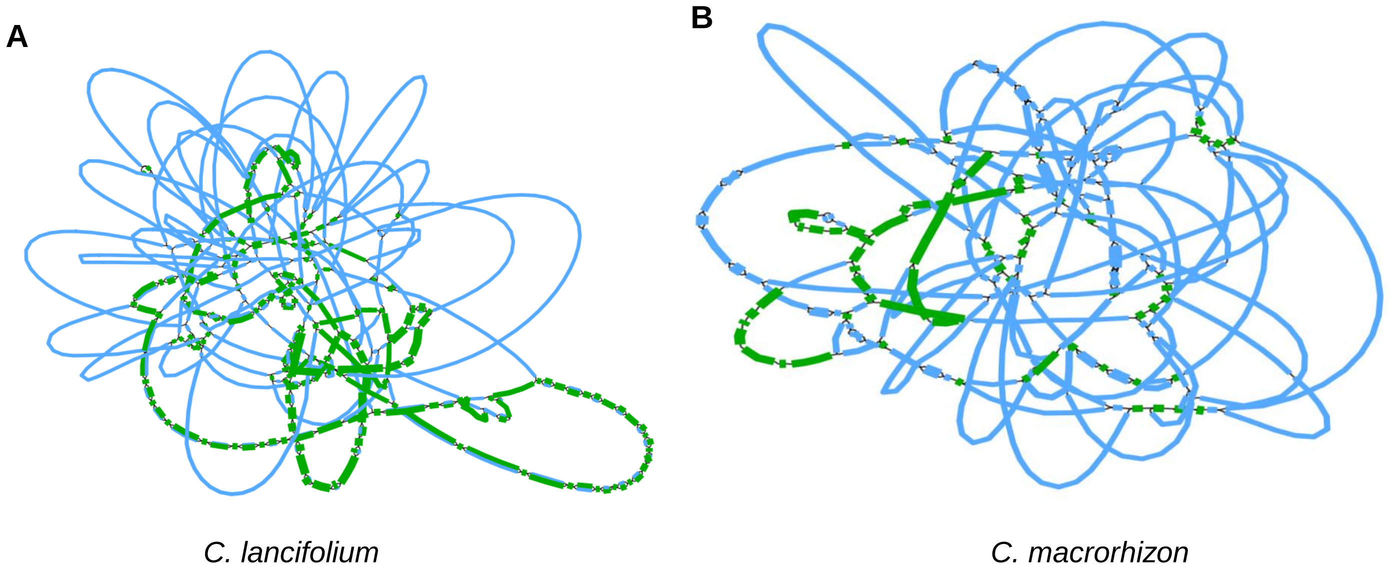

Fig. S3 | Read coverage of the *C. lancifolium* and *C. macrorhizon* mitogenomes. A) Read mapping plot of the *C. lancifolium* mitogenome. B) Read mapping plot of the *C. macrorhizon* mitogenome.

A

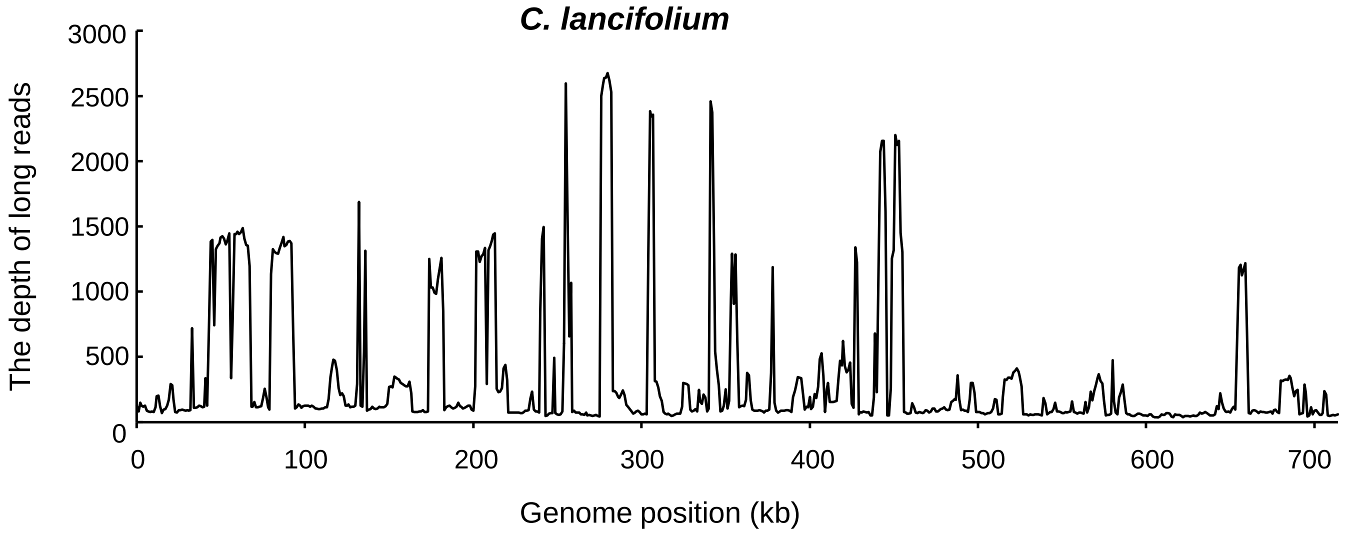

B

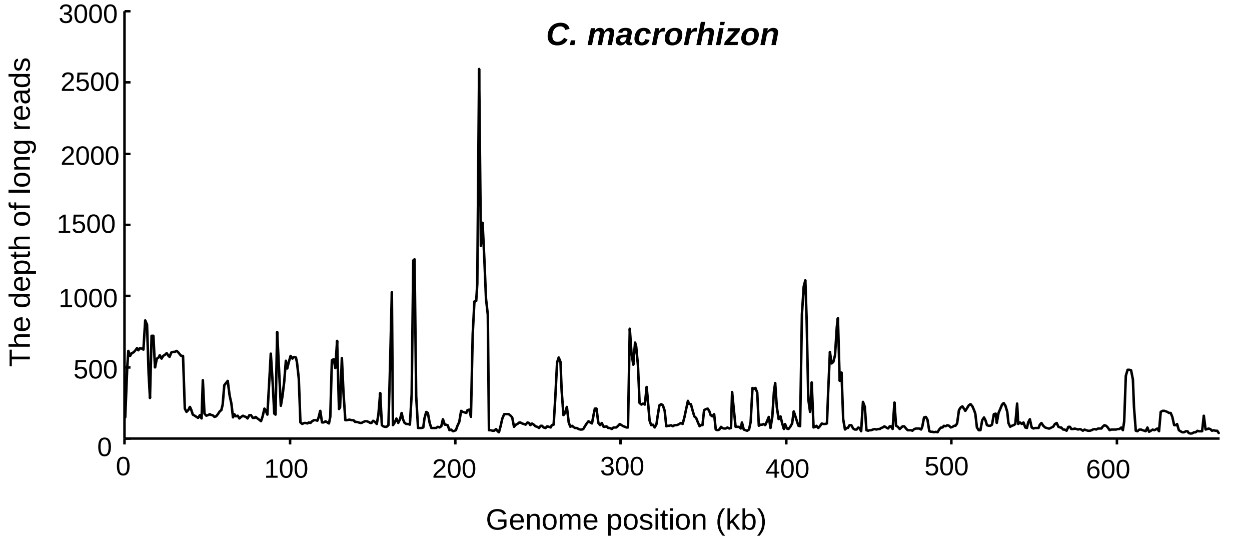

Fig. S4 | Evolution of boundary sequences of *C. lancifolium* repeats. A) Alignment of boundary sequences surrounding a 212 bp conserved block of 18 homologous repeats. B) Alignment of boundary sequences surrounding a 149 bp conserved block of 6 homologous repeats.

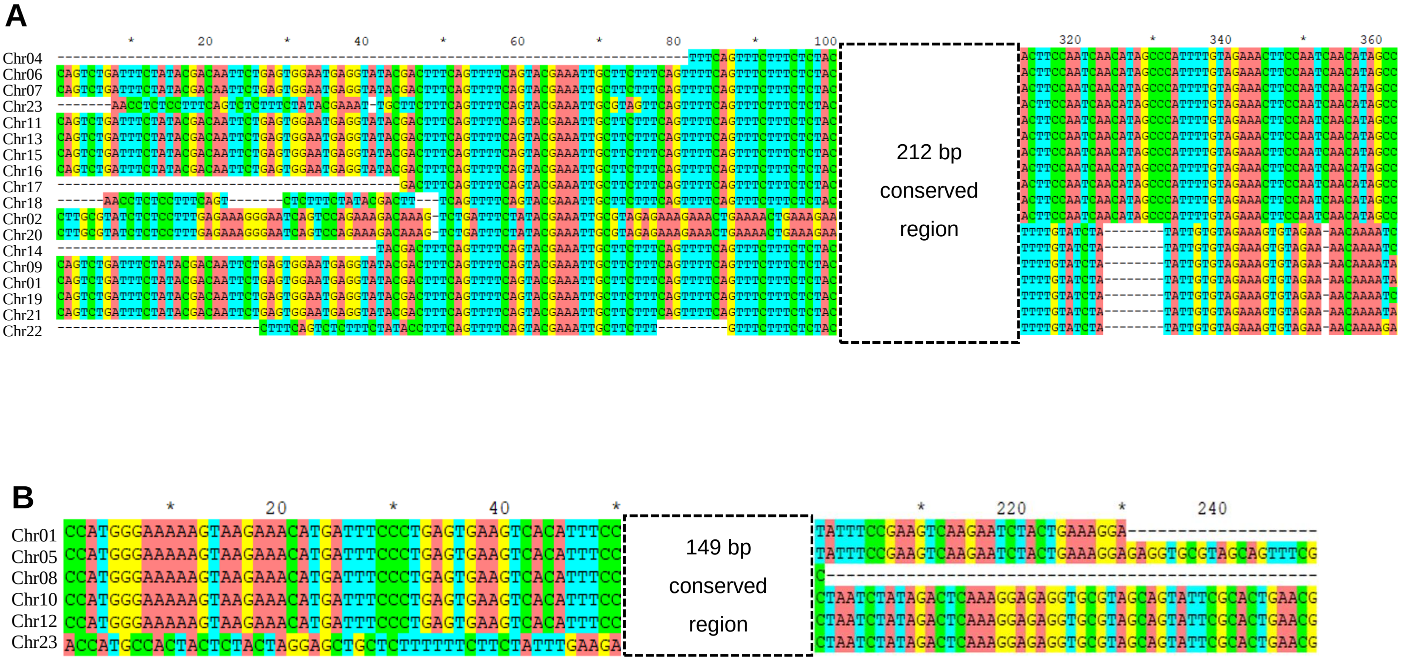

Fig. S5 | Evolution of boundary sequences of *C. macrorhizon* repeats. A) Alignment of boundary sequences surrounding a 140 bp conserved block of 19 homologous repeats. B) Alignment of boundary sequences surrounding a 149 bp conserved block of 6 homologous repeats.

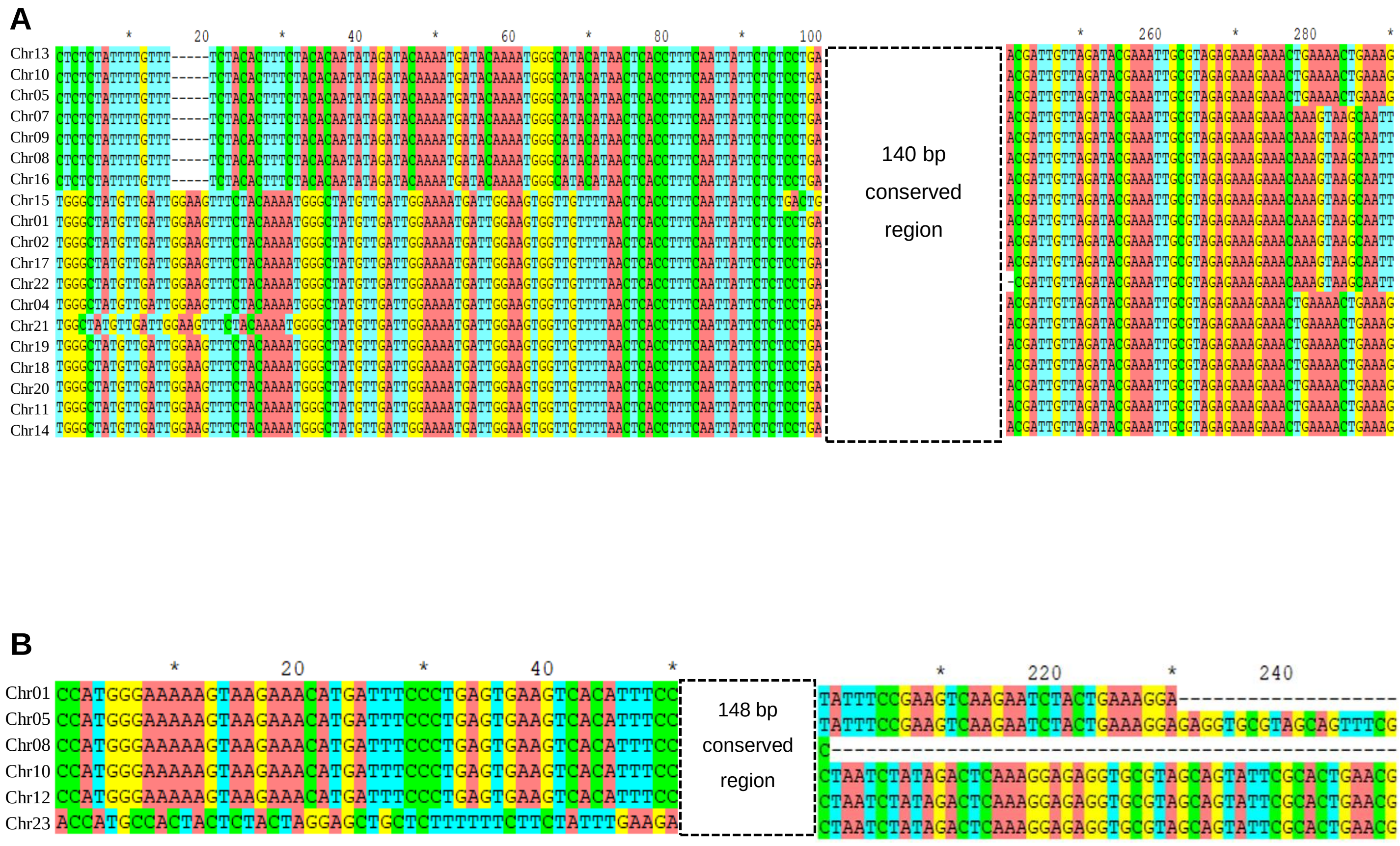

Fig. S6 | Identification and distribution of mitochondrial DNA of plastid origin (MIPT). A) MIPT sequences identified in chromosomes of the *C. lancifolium* mitogenome. B) MIPT sequences identified in chromosomes of the *C. macrorhizon* mitogenome. CP: chloroplast; Chr: chromosome. Red lines link homologous sequences with > 95% similarity and blue lines link homologous sequence with >90% similarity.

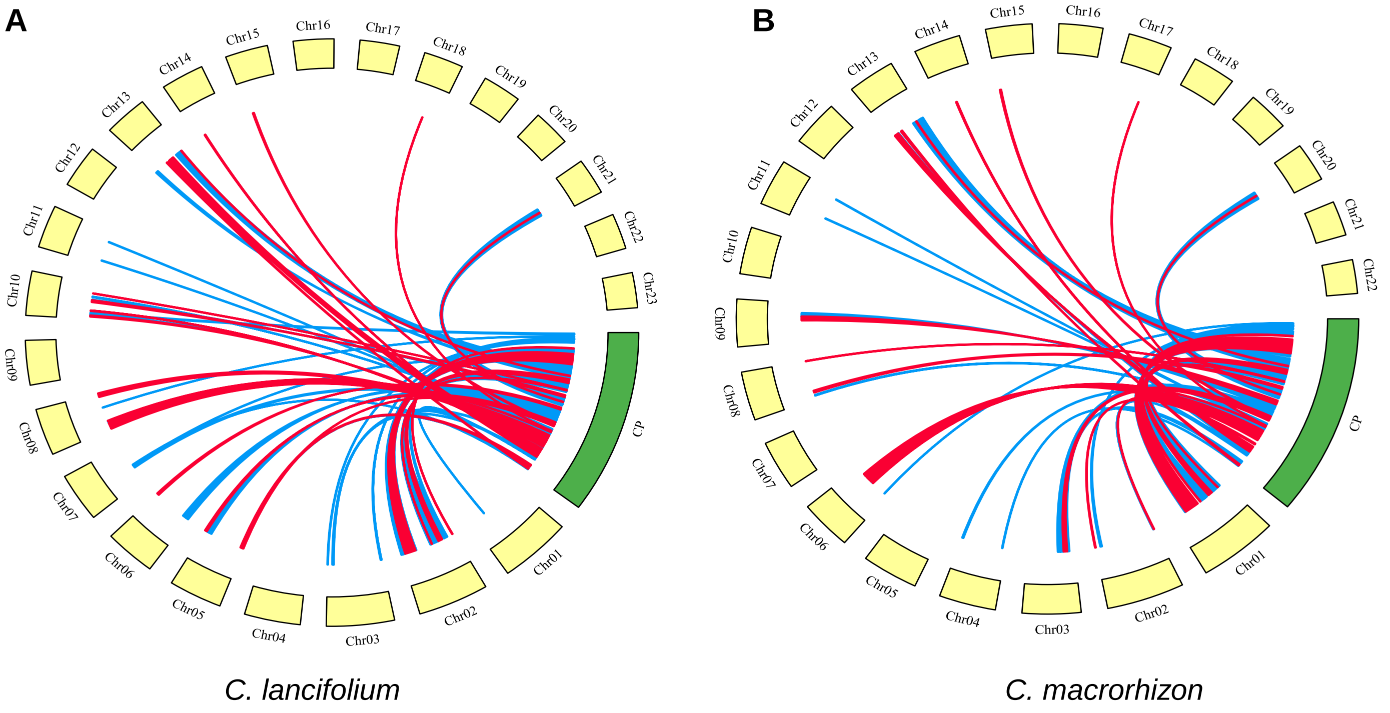

Supplement: Supplementary file 1 [file genes-14-00864-s001.zip › SupplementaryFigures.pdf]
